# Filth Annual Report of the State Commissioner in Lunacy

**Published:** 1878-08

**Authors:** 


*Fifth Annual Report of the State Commissioner in Lunacy.* Albany: 1878.

The report of the Commissioner In Lunacy, presented to the Legislature in January last contains much interesting information and is in a concise form, a brief and well digested review of the condition of the Insane in the State of New York.

In view of the growing disposition to care for the chronic insane in county asylums, the commissioner wisely recommends the appointment of a non-partisan board of managers, with a long term of office, to secure the benefit of experience, rather than the present method of frequent changes of management due to political influence and hence having most of the undesirable features of political management.

Dr. Ordronaux also calls attention to the need of a State Epileptic Asylum. In the various State and County Asylums there were reported last year 557 epileptics, a class of patients particularly undesirable for association with the Insane.

Some interesting statistics are given in the report. In the County Asylums we find that the cost of keeping the insane varies from ninety cents to three dollars per week. Otsego County reaching the extremely low limit and the Counties of Essex, Montgomery and Westchester reaching the higher one.

The weekly cost of maintenance of pauper patients in other institutions is as follows:

| | |
|---|---|
| New York County Idiot Asylum,..... .................. | $3.15. |
| State Asylum for Idiots, Syracuse,...................... | 4.06. |

| | |
|---|---|
| State Lunatic Asylum, Utica,........ .................. | 4.00. |
| Hudson River State Hospital, Poughkeepsie,............. | 5.50. |
| Willard Asylum, (Chronic),............................. | 2.88. |
| State Homœopathic Asylum, Middletown,............... | 4.50. |
| State Asylum for Insane Criminals,..................... | 4.00. |

Some excellent recommendations are contained in the report, especially in reference to the care of the chronic insane, and the State Inebriate Asylum. We regret that we have not room for a more extended notice. The work which the Commissioner is intended to perform is an extensive and important one and no one could do it more intelligently or conscientiously than the present excellent and genial Commissioner, Doctor Ordronaux.

